# REM sleep deprivation induces endothelial dysfunction and hypertension in middle-aged rats: Roles of the eNOS/NO/cGMP pathway and supplementation with L-arginine

**DOI:** 10.1371/journal.pone.0182746

**Published:** 2017-08-15

**Authors:** Jiaye Jiang, Zhongyuan Gan, Yuan Li, Wenqi Zhao, Hanqing Li, Jian-Pu Zheng, Yan Ke

**Affiliations:** Experimental Center for Teaching and Learning, Shanghai University of Traditional Chinese Medicine, Shanghai, PR China; Universidad Francisco de Vitoria, SPAIN

## Abstract

Sleep loss can induce or aggravate the development of cardiovascular and cerebrovascular diseases. However, the molecular mechanism underlying this phenomenon is poorly understood. The present study was designed to investigate the effects of REM sleep deprivation on blood pressure in rats and the underlying mechanisms of these effects. After Sprague-Dawley rats were subjected to REM sleep deprivation for 5 days, their blood pressures and endothelial function were measured. In addition, one group of rats was given continuous access to L-arginine supplementation (2% in distilled water) for the 5 days before and the 5 days of REM sleep deprivation to reverse sleep deprivation-induced pathological changes. The results showed that REM sleep deprivation decreased body weight, increased blood pressure, and impaired endothelial function of the aortas in middle-aged rats but not young rats. Moreover, nitric oxide (NO) and cyclic guanosine monophosphate (cGMP) concentrations as well as endothelial NO synthase (eNOS) phosphorylation in the aorta were decreased by REM sleep deprivation. Supplementation with L-arginine could protect against REM sleep deprivation-induced hypertension, endothelial dysfunction, and damage to the eNOS/NO/cGMP signaling pathway. The results of the present study suggested that REM sleep deprivation caused endothelial dysfunction and hypertension in middle-aged rats via the eNOS/NO/cGMP pathway and that these pathological changes could be inhibited via L-arginine supplementation. The present study provides a new strategy to inhibit the signaling pathways involved in insomnia-induced or insomnia-enhanced cardiovascular diseases.

## Introduction

Sleep is essential for an individual's mental, emotional, and physiological well-being. Insufficient sleep is prevalent in the population and is associated with cardiometabolic health outcomes[1]. It has been shown that insomnia with objective short sleep duration is associated with a high risk for hypertension[2,3], and the pathophysiological mechanisms underlying this association may relate to inappropriate arousal due to the overactivation of stress system functions[3,4].

Endothelial dysfunction, which manifests as a reduced vasodilating response to endothelial stimuli, has prognostic significance and serves as an early indicator of the development of various vascular diseases, including hypertension[5,6]. It has been reported that sleep deprivation in animals or healthy subjects can cause vascular dysfunction[7–9]; however, the mechanism underlying this phenomenon remains poorly understood. The present study was designed to investigate the molecular mechanisms of REM sleep deprivation-induced hypertension and endothelial dysfunction. The results showed that REM sleep deprivation can impair nitric oxide (NO) signaling and cause endothelial dysfunction and hypertension in rats and that supplementation with L-arginine can suppress the pathological changes induced by REM sleep deprivation.

## Materials and methods

### Animals

Six-week-old (young) and 24-week-old (middle-aged) male Sprague-Dawley rats were purchased from Shanghai Slack Laboratory Animal Co., Ltd. (Shanghai, China). All of the animals were housed in individual cages on a 12 h light-dark cycle in a room with temperature and humidity control and were allowed access to standard rat chow and distilled water ad libitum. All experimental procedures were conducted in accordance with the National Institutes of Health Guide for the Care and Use of Laboratory Animals and were approved by the ethics committee of Shanghai University of Traditional Chinese Medicine. After 1 week of accommodation to environmental conditions, animals were used for experiments.

### REM sleep deprivation

Animals were deprived of sleep via the disk-over-water method, with certain modifications[10]. Briefly, animals were continuously kept on a small raised platform (with a diameter of 6.0 cm for young rats and 6.5 cm for middle-aged rats) surrounded by water up to 1 cm beneath the platform surface for 5 days. When they reached the paradoxical phase of sleep, muscle atonia caused them to fall into the water and awaken. Control rats were maintained on a larger platform (with a diameter of 15 cm) in a similar environment. Rats supplemented with L-arginine were continuously provided with access to L-arginine (2% in distilled water) for 5 days before REM sleep deprivation. Subsequently, these animals were continuously provided with access to L-arginine or vehicle (distilled water) for the 5 days of REM sleep deprivation. Food and water were available ad libitum through a grid placed atop the water tank. Following REM sleep deprivation, rats' blood pressures were measured, and the rats were sacrificed. Their aortas were carefully collected for further examination.

### Blood pressure measurement

Rats were anesthetized with isoflurane. Systolic blood pressure (SBP) was measured using tail-cuff plethysmography (TCP), as described previously[11]. TCP was performed using an automated approach (Alcott Biotech, Shanghai, China). TCP values were determined by averaging at least five consecutive measurements obtained after signal stabilization.

### Myograph study

Rats were sacrificed, and the descending thoracic aorta was harvested. Vasorelaxation was measured using a 620M myograph system (DMT, Aarhus N, Denmark)[12]. Briefly, artery rings were suspended in bicarbonate buffer solution at 37°C and continuously aerated with 95% O_2_ and 5% CO_2_ for the recording of isometric tension in organ chambers. First, they were stretched to a resting tension of 2 g and allowed to equilibrate for a period of at least 60 min. Tension was readjusted when necessary, and the bath fluid was changed every 20 min. After they had stabilized, rings were exposed twice to 60 mM KCl to obtain reference contractions. Thereafter, they were contracted using phenylephrine (PE, 1 μM) and relaxed using cumulative concentrations of acetylcholine (ACh) to investigate endothelium-dependent vasodilatation in the absence or presence of different inhibitors. The utilized inhibitors included L-NAME (a nitric oxide synthase inhibitor), indomethacin (a cyclooxygenase inhibitor), and TEA (an inhibitor of endothelium-derived hyperpolarizing factor (EDHF))[11,13]. The dilation response to ACh was presented as the percentage of the contractile response induced by PE, and the maximal relaxation (Emax) was calculated for statistical analysis.

### NO assay

NO production in situ is difficult to detect because of its rapid decay (within seconds) in physiological systems. However, NO levels can be evaluated by measuring nitrates and nitrites (NOx), which are metabolites of NO. In this study, we measured the accumulation of total nitrites in samples using the Total Nitric Oxide Assay Kit (Beyotime Biotech, Shanghai, China). After color development at room temperature, samples’ absorbances were measured on a microplate reader at a wavelength of 540 nm. Sodium nitrite (NaNO_2_) was used as an external standard, and NO levels in samples were expressed as μM nitrites/g tissue.

### Enzyme-linked immunosorbent assay

Cyclic guanosine monophosphate (cGMP) concentrations in lysates of rat aorta tissue were determined using an enzyme-linked immunosorbent assay (ELISA) cGMP detection kit (R&D Systems, Minneapolis, MN, USA) in accordance with the manufacturer’s instructions.

### Protein extraction and western blotting

Aortas were lysed in a buffer [1% phenylmethylsulfonyl fluoride (PMSF) and 0.004% complete inhibitor] and homogenized on ice. The supernatant was collected after 10 min of centrifugation at 12,000×*g* and 4°C, and protein concentration was determined using a BCA Protein Assay Kit (Pierce, Rockford, IL, USA). Equal quantities of proteins (30 μg) were separated via 10% sodium dodecyl sulfate (SDS) polyacrylamide gel electrophoresis (SDS-PAGE) and transferred to nitrocellulose membranes (Bio-Rad Laboratories, Hercules, CA, USA). After blocking, each membrane was incubated with primary antibodies at 4°C overnight. The present study utilized antibodies against endothelial nitric oxide synthase (eNOS) (1:500 dilution; BD Transduction Laboratories^™^; 610297), phospho-eNOS (p-eNOS) (S1177; 1:500 dilution; Abcam; ab51038), and glyceraldehyde-3-phosphate dehydrogenase (GAPDH) (1:5000 dilution; Kangchen; KC-5G5). After three washes with Tris (plus Tween 20) buffer, membranes were incubated with horseradish peroxidase (HRP)-labeled goat anti-rabbit or anti-mouse IgG for 1 h at room temperature. Protein bands were detected using enhanced chemiluminescence (ECL) reagents. Chemiluminescent signals were detected and analyzed using the ChemiDoc XRS Imaging System (Tanon, Shanghai, China).

### Statistical analysis

All data were expressed as means±SEM, and *n* refers to the number of rats. SPSS 18.0 (SPSS Inc, Chicago, IL, USA) was used for comparisons of multiple groups via one-way analysis of variance (ANOVA) and LSD tests. Differences were considered statistically significant if *P*<0.05.

## Results

### Effects of REM sleep deprivation on body weight and blood pressure

**Fig 1:**
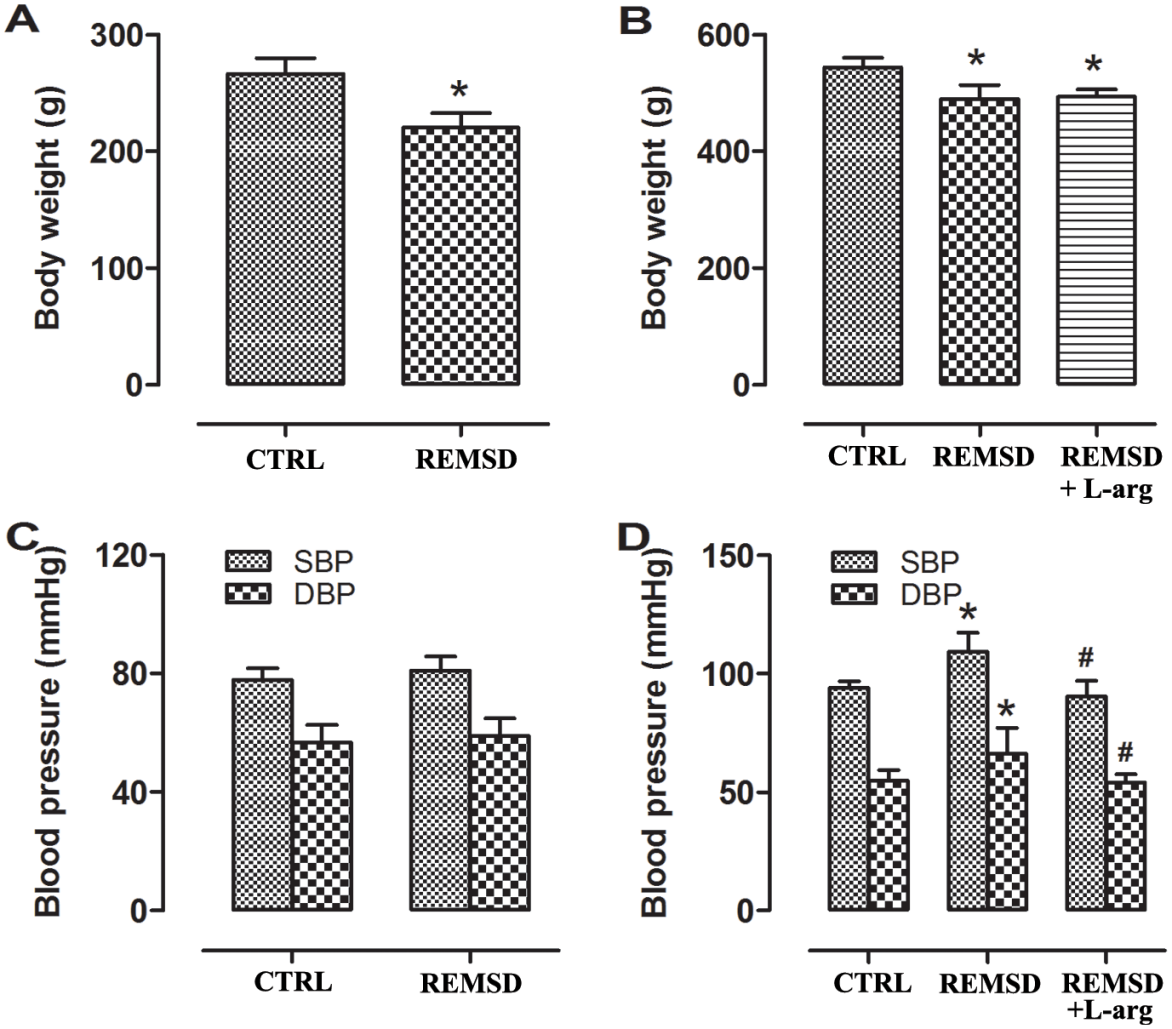
Body weights and blood pressures with or without REM sleep deprivation (REMSD). (A)Body weights of young-aged rats.(B)Body weights of middle-aged rats (C)Blood pressures of young-aged rats.(D)Blood pressures of middle-aged rats.Data were expressed as means±SEM; n = 6 rats per study group. *P<0.05 *vs*. CTRL; ^#^*P*<0.05 *vs*.REMSD. CTRL refers to rats not subjected to REM sleep deprivation. SBP, systolic blood pressure; DBP, diastolic blood pressure; L-arg, L-arginine.

REM sleep deprivation caused significant reductions in body weight in young and middle-aged rats (Fig 1A and 1B). This sleep deprivation also increased the blood pressure of middle-aged rats, with increases of 16% and 18% in systolic and diastolic blood pressure, respectively (Fig 1D). However, the blood pressure of young rats was not changed after 5 days of sleep deprivation (Fig 1C).

### Effects of REM sleep deprivation on endothelial function

**Fig 2:**
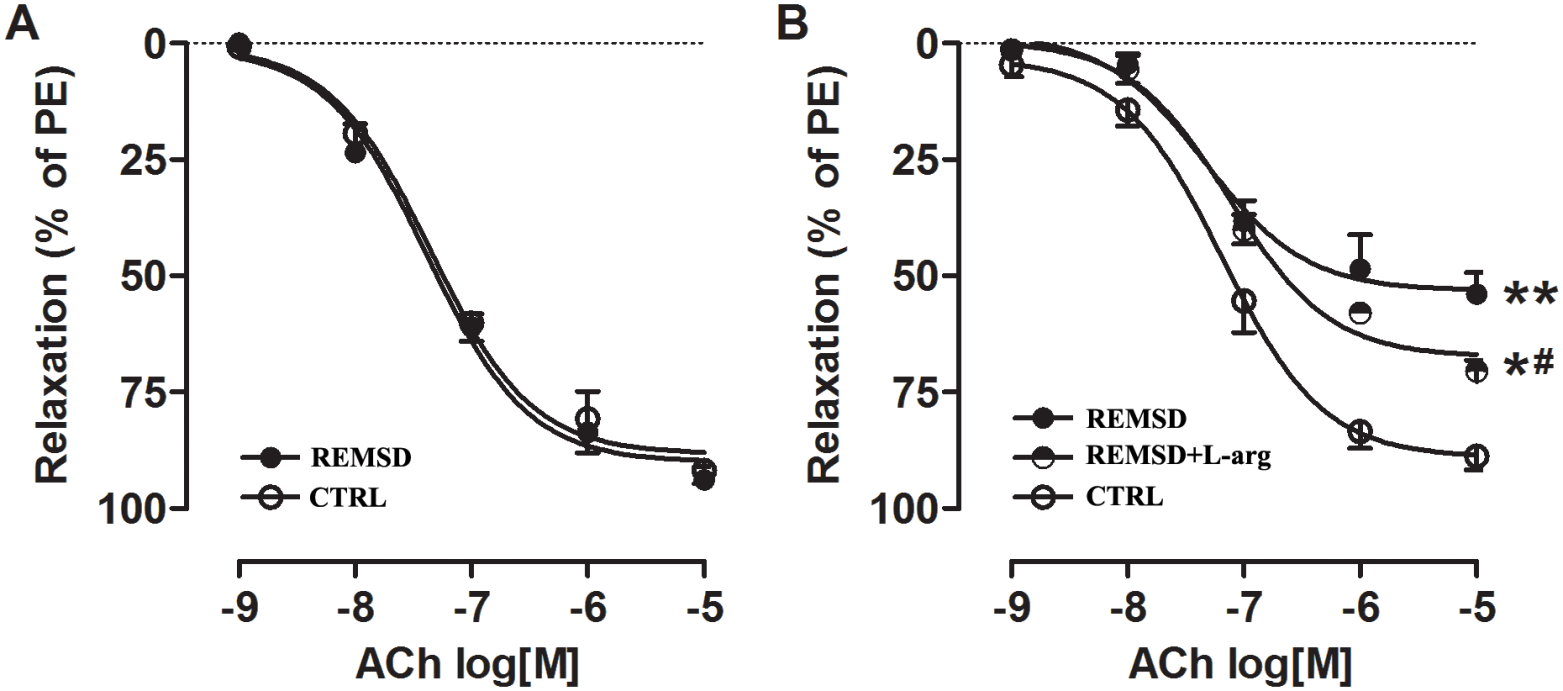
Acetylcholine (ACh)-induced vasorelaxation of the aortas of rats. (A)ACh-induced vasorelaxation of young-aged rats.(B)ACh-induced vasorelaxation of middle-aged.The dilation response to ACh is presented as the percentage of the contractile response induced by phenylephrine (PE, 10^−6^ M). Each point represents a mean±SEM; n = 6 rats per study group. *E_max_ significantly different from that of CTRL (*P<0.05 and **P<0.01). ^#^E_max_ significantly different from that of REMSD (^#^*P*<0.05). CTRL, control rats without REM sleep deprivation; REMSD,REM sleep deprivation; L-arg, L-arginine.

**Fig 3:**
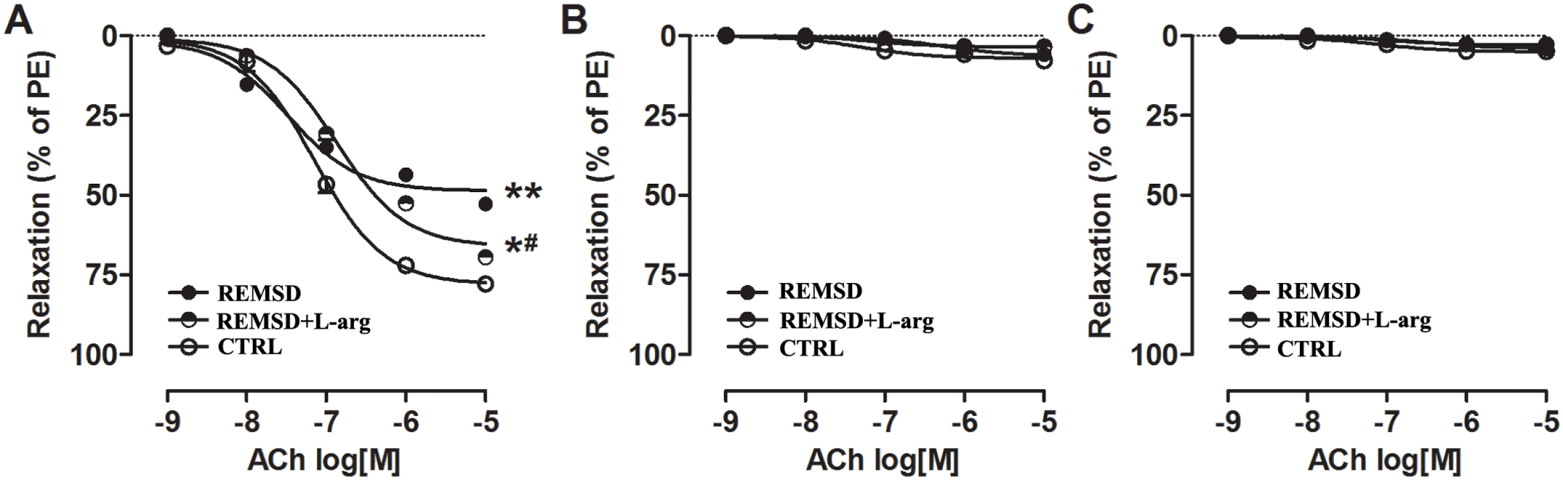
Different channel mediated vasorelaxation of the aortas of middle-aged rats. (A)NO-mediated vasorelaxation.NO-mediated relaxation was determined in the presence of 10 μM indomethacin and 1 μM TEA to block PGI_2_ and EDHF, respectively. (B)PGI_2_-mediated vasorelaxation.PGI_2_-mediated relaxation was evaluated with 100 μM L-NAME and 1 μM TEA to block NO and EDHF, respectively.(C)EDHF-mediated vasorelaxation.EDHF-mediated relaxation was determined in the presence of 100 μM L-NAME and 10 μM indomethacin to block NO and PGI_2_, respectively. Each point represents a mean±SEM; n = 6 rats per study group. *E_max_ significantly different from that of CTRL (*P<0.05). ^#^E_max_ significantly different from that of REMSD (^#^*P*<0.05). CTRL, control rats without REM sleep deprivation; REMSD, REM sleep deprivation; L-arg, L-arginine.

REM sleep deprivation did not affect ACh-mediated vasodilatation in the aortas of young rats (Fig 2A). However, the endothelial function of middle-aged rats was significantly damaged by REM sleep deprivation (Fig 2B). To date, the major dilatory mediators released by ACh that have been characterized are NO, prostacyclin (PGI_2_) and EDHF[13]. To investigate which factor mediated relaxation that was repaired by REM sleep deprivation, L-NAME (100 μM), indomethacin (10 μM) and TEA (1 μM) were used to block NO, PGI_2_ and EDHF, respectively. As shown in Fig 3A, REM sleep deprivation caused significant impairment of NO-mediated vasodilation of the rat aorta. However, aorta relaxation mediated by PGI_2_ or EDHF was negligible in both groups and was unchanged by REM sleep deprivation (Fig 3B and 3C).

### Effects of REM sleep deprivation on NO and cGMP concentrations in middle-aged rats

**Fig 4:**
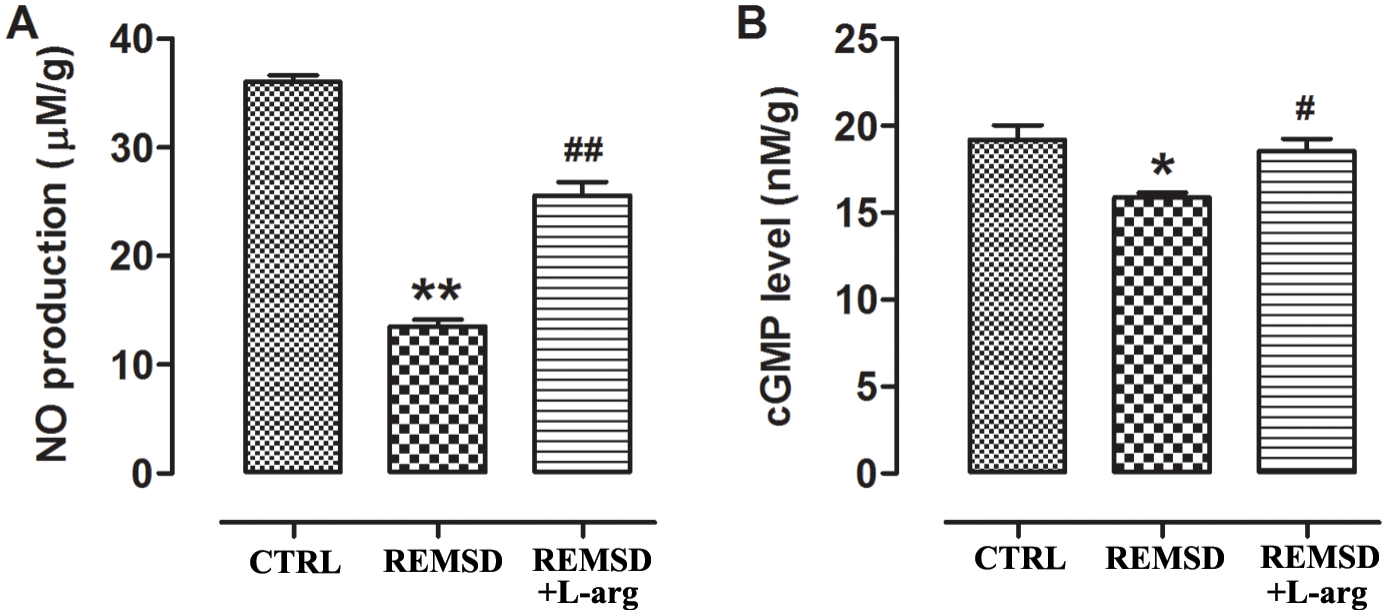
NO production and cGMP concentration in the aortas of middle-aged rats. (A)NO production. NO in the aorta was measured using the Greiss reagent. (B) cGMP concentration,cGMP in the aorta was measured using ELISA. Data were expressed as means±SEM; *n* = 6. *P<0.05 and **P<0.01 *vs*. CTRL; ^#^*P*<0.05 and ^##^*P*<0.01 *vs*. REMSD. CTRL, control rats without REM sleep deprivation; REMSD, REM sleep deprivation; L-arg, L-arginine.

As shown in Fig 4, REM sleep deprivation decreased NO production and cGMP levels in the aortas of middle-aged rats.

### Effects of REM sleep deprivation on eNOS expression in middle-aged rats

**Fig 5:**
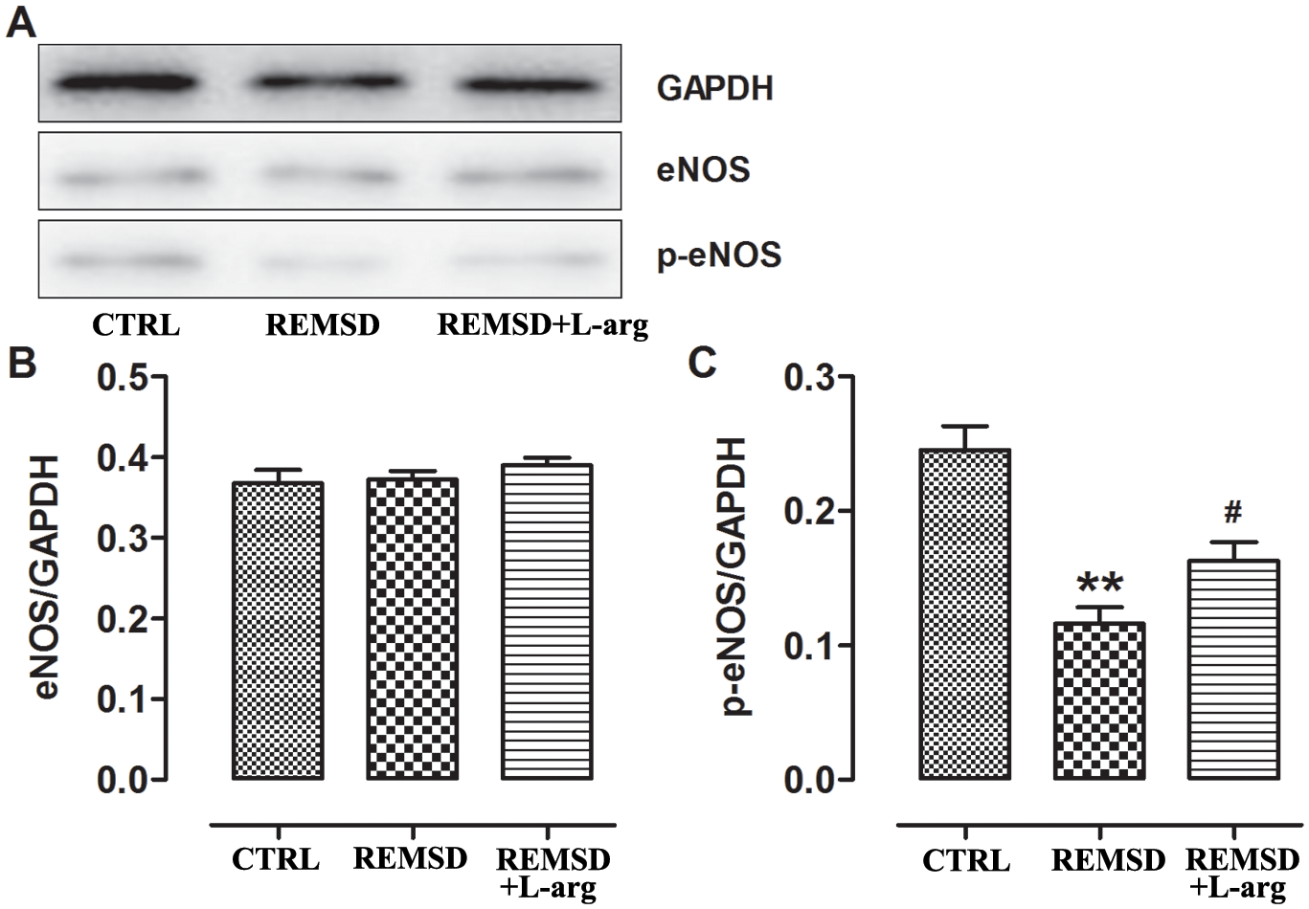
Protein expression and phosphorylation of eNOS in the aortas of middle-aged rats. Protein expression levels in the aorta were measured via (A)western blotting. (B and C)Chemiluminescent signals were detected and analyzed using the ChemiDoc XRS Imaging System.Data were expressed as means±SEM; *n* = 6. *P<0.05 and **P<0.01 *vs*. CTRL; ^#^*P*<0.05 *vs*. REMSD. CTRL, control rats without REM sleep deprivation; REMSD, REM sleep deprivation; L-arg, L-arginine.

Western blotting revealed decreased expression of the phosphorylated eNOS protein in REM sleep-deprived rats. However, the overall level of eNOS protein was not significantly affected by REM sleep deprivation (Fig 5).

### Effects of L-arginine supplementation

Given that the eNOS/NO/cGMP signaling pathway was hindered by REM sleep deprivation, rats were provided with drinking water supplemented with L-arginine to improve this pathway and ameliorate endothelial dysfunction. The results showed that L-arginine decreased blood pressure (Fig 1D), improved NO-mediated vasorelaxation (Figs 2B and 3A), raised NO and cGMP levels in the aorta (Fig 4), and increased eNOS phosphorylation (Fig 5).

## Discussion

The present study showed that REM sleep deprivation damaged the eNOS/NO/cGMP signaling pathway and caused endothelial dysfunction and hypertension. L-arginine supplementation could protect against these changes induced by REM sleep deprivation; this finding might give rise to a new interventional strategy for sleep loss-induced hypertension or other cardiovascular diseases.

Sleep is divided into the following two broad types in mammals and birds: REM sleep and non-REM sleep. During REM sleep, muscles become completely paralyzed and unresponsive, a state known as atonia. The disk-over-water method used in the present study is an approach for inducing REM sleep deprivation[14]. REM sleep deprivation of rats for 5 days increased blood pressure in middle-aged rats but not young rats (Fig 1C and 1D), suggesting that this reaction might be age-dependent. The present results were consistent with clinical studies indicating that a short sleep duration was associated with an increased risk of hypertension in middle-aged and elderly subjects[15–17], suggesting that elderly people are particularly vulnerable to sleep loss or insomnia.

Endothelial dysfunction, defined as a reduction in the ability of the endothelium to transmit a vasodilatory influence on blood flow, serves as an early indicator of the development of hypertension[5,6]. We studied whether endothelial dysfunction also existed in REM sleep deprivation-induced hypertension. The results showed that endothelium-dependent vasorelaxation was decreased significantly in middle-aged rats with REM sleep deprivation (Fig 2); this change was due to impaired NO production (Fig 3A).

In the blood vessel wall, NO is produced mainly from L-arginine by the enzyme eNOS and regulates the degree of contraction of vascular smooth muscle cells mainly by stimulating soluble guanylyl cyclase (sGC) to produce cGMP[18]. In the present study, eNOS phosphorylation, NO, and cGMP levels in the aorta were decreased by sleep deprivation (Figs 4 and 5), indicating damage to the eNOS/NO/cGMP signaling pathway in middle-aged rats subjected to REM sleep deprivation.

Given that exogenous L-arginine supplementation can ameliorate the development of hypertension in rats[19–21], we next observed the effects of L-arginine on REM sleep deprivation-induced pathological changes in middle-aged rats. In this study, we treated REM sleep deprivation with L-arginine in drinking water. As expected, L-arginine supplementation could increase eNOS phosphorylation (Fig 5), augment NO and cGMP production (Fig 4), improve NO-mediated vasodilation (Figs 2B and 3A), and consequently decrease blood pressure (Fig 1D). Interestingly, L-arginine supplementation can protect against REM sleep deprivation-induced endothelial dysfunction and hypertension. In addition, the study results also indicate that endothelial dysfunction due to damaged NO bioavailability contributes to REM sleep deprivation-induced hypertension.

Given that there are many adverse side effects of the long-term usage of insomnia medications to improve insomnia or increase sleep duration[22], it is difficult to suppress sleep loss-induced adverse effects on the cardiovascular system. The present study provides a new strategy for inhibiting the signaling pathways that contribute to insomnia-induced or insomnia-enhanced cardiovascular diseases.

However, the present study was performed in SD rats. It is unclear if other rat strains also have similar response to REM sleep deprivation. Different rat strains may have different responses given different genetic makeup. Further study is needed to understand this question.

## Supporting information

S1 File Data availability statement. Important data of figures in the article.
(PDF)

S2 File ARRIVE guidelines checklist. (PDF)

## References

[1] Altman NG, Izci-Balserak B, Schopfer E, Jackson N, Rattanaumpawan P, Gehrman PR, et al Sleep duration versus sleep insufficiency as predictors of cardiometabolic health outcomes. Sleep Med. 2012;13: 1261–1270. doi: 10.1016/j.sleep.2012.08.005 23141932 10.1016/j.sleep.2012.08.005 PMC3527631

[2] Vgontzas AN, Liao D, Bixler EO, Chrousos GP and Vela-Bueno A. Insomnia with objective short sleep duration is associated with a high risk for hypertension. Sleep. 2009;32: 491–497. 19413143 10.1093/sleep/32.4.491 PMC2663863

[3] Li Y, Vgontzas AN, Fernandez-Mendoza J, Bixler EO, Sun YF, Zhou JY, et al Insomnia with physiological hyperarousal is associated with hypertension. Hypertension. 2015;65: 644–650. doi: 10.1161/HYPERTENSIONAHA.114.04604 25624338 10.1161/HYPERTENSIONAHA.114.04604

[4] Palagini L, Maria Bruno R, Gemignani A, Baglioni C, Ghiadoni L, Dieter R. Sleep Loss and Hypertension: A Systematic Review. Curr Pharm Des. 2013;19: 2409–2419. 23173590 10.2174/1381612811319130009

[5] Yolanda M, Silvia L, and Eduardo N. Reactivity of the aorta and mesenteric resistance arteries from the obese spontaneously hypertensive rat: effects of glitazones. Am J Physiol Heart Circ Physiol.2011;301: H1319–1330. doi: 10.1152/ajpheart.01280.2010 21784989 10.1152/ajpheart.01280.2010

[6] Kensuke E. Clinical Importance of Endothelial Function in Arteriosclerosis and Ischemic Heart Disease. Circ J. 2002;66: 529–533. 12074266 10.1253/circj.66.529

[7] Sauvet F, Florence G, Van Beers P, Drogou C, Lagrume C, Chaumes C, et al Total sleep deprivation alters endothelial function in rats: a nonsympathetic mechanism. Sleep. 2014;37: 465–473. doi: 10.5665/sleep.3476 24587568 10.5665/sleep.3476 PMC3920311

[8] Calvin AD, Covassin N, Kremers WK, Adachi T, Macedo P, Albuquerque FN, et al Experimental sleep restriction causes endothelial dysfunction in healthy humans. J Am Heart Assoc. 2014;3: e001143 doi: 10.1161/JAHA.114.001143 25424573 10.1161/JAHA.114.001143 PMC4338700

[9] Sauvet F, Leftheriotis G, Gomez-Merino D, Langrume C, Drogou C, Beers PV, et al Effect of acute sleep deprivation on vascular function in healthy subjects. J Appl Physiol (1985). 2010;108: 68–75. 19910332 10.1152/japplphysiol.00851.2009

[10] Gopalakrishnan A, Ji LL, Cirelli C. Sleep deprivation and cellular responses to oxidative stress. Sleep. 2004;27: 27–35. 14998234 10.1093/sleep/27.1.27

[11] Jiang J, Zheng JP, Li Y, Gan ZY, Jiang YB, Huang D, et al Differential contribution of endothelium-derived relaxing factors to vascular reactivity in conduit and resistance arteries from normotensive and hypertensive rats. Clin Exp Hypertens. 2016;38: 393–398. doi: 10.3109/10641963.2016.1148155 27159544 10.3109/10641963.2016.1148155

[12] Zheng JP, Cheng ZA, Jiang JJ, Ke Y, Liu ZJ. Cyclosporin A upregulates ETB receptor in vascular smooth muscle via activation of mitogen-activating protein kinases and NF-kappaB pathways. Toxicol Lett. 2015;235: 1–7. doi: 10.1016/j.toxlet.2015.03.004 25772258 10.1016/j.toxlet.2015.03.004

[13] Alm R, Lars E, Malmsjö M. Organ culture: a new model for vascular endothelium dysfunction. BMC Cardiovascular Disord. 2002;2: 1–7. 10.1186/1471-2261-2-8 PMC113257 12019023

[14] Villafuerte G, Miguel-Puga A, Rodriguez EM, Machado S, Manjarrez E, Arias-Carrion O. Sleep deprivation and oxidative stress in animal models: a systematic review. Oxid Med Cell Longev. 2015;2015: 234952 doi: 10.1155/2015/234952 25945148 10.1155/2015/234952 PMC4402503

[15] Sun XM, Yao S, Hu SJ, Liu ZY, Yang YJ, Yuan ZY, et al Short sleep duration is associated with increased risk of pre-hypertension and hypertension in Chinese early middle-aged females. Sleep Breath. 2016;20: 1355–1362. doi: 10.1007/s11325-016-1392-2 27491292 10.1007/s11325-016-1392-2

[16] Guo J, Fei Y, Li JQ, Zhang LS, Luo Q, Chen GD. Gender- and age-specific associations between sleep duration and prevalent hypertension in middle-aged and elderly Chinese: a cross-sectional study from CHARLS 2011–2012. BMJ open. 2016;6: e011770 doi: 10.1136/bmjopen-2016-011770 27601494 10.1136/bmjopen-2016-011770 PMC5020843

[17] Fung MM, Peters K, Redline S, Ziegler MG, Ancoli-Israel S, Barrett-Connor E, et al Decreased slow wave sleep increases risk of developing hypertension in elderly men. Hypertension. 2011;58: 596–603. doi: 10.1161/HYPERTENSIONAHA.111.174409 21876072 10.1161/HYPERTENSIONAHA.111.174409 PMC3176739

[18] Evora PRB, Evora PM, Celotto AC, Rodrigues AJ, Joviliano EE. Cardiovascular Therapeutics Targets on the NO—sGC—cGMP Signaling Pathway: A Critical Overview. Current Drug Targets. 2012;13: 1207–1214. 22716077 10.2174/138945012802002348

[19] Chen PY, Sanders PW. L-arginine abrogates salt-sensitive hypertension in Dahl/Rapp rats. J Clin Invest. 1991;88: 1559–1567. doi: 10.1172/JCI115467 1658045 10.1172/JCI115467 PMC295672

[20] Katoh T, Takahashi K, Klahr S, Reyes AA and Badr KF. Dietary supplementation with L-arginine ameliorates glomerular hypertension in rats with subtotal nephrectomy. J Am Soc Nephrol. 1994;4: 1690–1694. 8011979 10.1681/ASN.V491690

[21] Rajapakse NW, Miguel CD, Das S, Mattson DL. Exogenous L-arginine ameliorates angiotensin II-induced hypertension and renal damage in rats. Hypertension. 2008;52: 1084–1090. doi: 10.1161/HYPERTENSIONAHA.108.114298 18981330 10.1161/HYPERTENSIONAHA.108.114298 PMC2680209

[22] Norman JL, Anderson SL. Novel class of medications, orexin receptor antagonists, in the treatment of insomnia—critical appraisal of suvorexant. Nat Sci Sleep. 2016;8: 239–247. doi: 10.2147/NSS.S76910 27471419 10.2147/NSS.S76910 PMC4948724

