# Supplementary material for: REM sleep deprivation induces endothelial dysfunction and hypertension in middle-aged rats: Roles of the eNOS/NO/cGMP pathway and supplementation with L-arginine
Source: PLoS One. 2017 Aug 15;12(8):e0182746. doi: 10.1371/journal.pone.0182746 (PMC5557538; doi:10.1371/journal.pone.0182746)
Supplement: S1 File — Important data of figures in the article. (PDF) [file pone.0182746.s001.pdf]

Table 1 Body weight (kg)

	young-aged rats	middle-aged rats
CTRL	266.25±3.5	544±16.41
REMSD	220±2.58*	489.28±24.05*
REMSD+L-arg		493.50±2.12*

*P<0.05 *vs*. CTRL; #P<0.05 *vs*. REMSD. CTRL refers to rats not subjected to REM sleep deprivation; REMSD: REM sleep deprivation; L-arg: L-arginine.

Table 2 Blood pressure (mmHg)

	young-aged rats		middle-aged rats	
	SBP	DBP	SBP	DBP
CTRL	77.8±3.91	56.6±6.01	94±2.84	54.98±4.31
REMSD	80.93±4.74	58.7±6.01	109.±8.08*	66.08.±11.06*
REMSD+L-arg			90.3±6.8#	54.13±3.39#

*P<0.05 *vs*. CTRL; #P<0.05 *vs*. REMSD. CTRL refers to rats not subjected to REM sleep deprivation; REMSD: REM sleep deprivation; L-arg: L-arginine. SBP: systolic blood pressure; DBP: diastolic blood pressure.

Table 3 Relaxation of PE（10^{-5} Ach）

	young-aged rats	middle-aged rats
CTRL	91.81±2.90	88.74±3.04
REMSD	93.78±2.66	53.89±4.59**
REMSD+L-arg		70.43±2.25*#

*Emax significantly different from that of CTRL (*P<0.05 and **P<0.01). #Emax significantly different from that of REMSD (#P<0.05). CTRL refers to rats not subjected to REM sleep deprivation; REMSD:REM sleep deprivation; L-arg: L-arginine.

Table 4 NO-mediated , PGI2-mediated or EDHF-mediated vasorelaxation of the aortas of middle-aged rats（10^{-5} Ach）

	NO-mediated	PGI2-mediated	EDHF-mediated
CTRL	77.84±0.92	8.80±0.07	8.80±0.07
REMSD	52.75±0.63**	2.88±0.79	4.05.±0.73
REMSD+L-arg	69.45±0.75*#	3.39±0.17	2.73±0.55

NO-mediated relaxation was determined in the presence of 10 μM indomethacin and 1 μM TEA to block PGI2 and EDHF, respectively. PGI2-mediated relaxation was evaluated with 100 μM L-NAME and 1 μM TEA to block NO and EDHF, respectively. EDHF-mediated relaxation was determined in the presence of 100 μM L-NAME and 10 μM indomethacin to block NO and PGI2, respectively. *Emax significantly different from that of CTRL (*P<0.05). #Emax significantly different from that of REMSD (#P<0.05). CTRL refers to rats not subjected to REM sleep deprivation; REMSD:REM sleep deprivation; L-arg: L-arginine.

Table 5 NO production and cGMP concentration in the aortas of middle-aged rats

	NO(μ M/g)	cGMP(nM/g)
CTRL	36.02±0.62	19.18±0.82
REMSD	13.53±0.61**	15.84±0.28*
REMSD+L-arg	25.54±1.25##	18.54±0.71#

*P<0.05 and **P<0.01 *vs*. CTRL; #P<0.05 and ##P<0.01 *vs*. REMSD. CTRL refers to rats not subjected to REM sleep deprivation; REMSD:REM sleep deprivation; L-arg: L-arginine.

Table 6 Protein expression and phosphorylation of eNOS in the aortas of middle-aged rats

	eNOS/GAPDH	p-eNOS/GAPDH
CTRL	0.37±0.02	0.25±0.04
REMSD	0.37±0.01	0.12±0.003**
REMSD+L-arg	0.41±0.01	$0.15±0.03^{\#}$

**P<0.01 *vs*. CTRL; $^{\#}P$<0.05 *vs*. REMSD. CTRL refers to rats not subjected to REM sleep deprivation; REMSD: REM sleep deprivation; L-arg: L-arginine.
