# Supplementary material for: REM sleep deprivation induces endothelial dysfunction and hypertension in middle-aged rats: Roles of the eNOS/NO/cGMP pathway and supplementation with L-arginine
Source: PLoS One. 2017 Aug 15;12(8):e0182746. doi: 10.1371/journal.pone.0182746 (PMC5557538; doi:10.1371/journal.pone.0182746)
Supplement: S2 File — (PDF) [file pone.0182746.s002.pdf]

	ITEM	RECOMMENDATION
Title	1	REM sleep deprivation induces endothelial dysfunction and hypertension in middle-aged rats: roles of the eNOS/NO/cGMP pathway and supplementation with L-arginine
Abstract	2	Sleep loss can induce or aggravate the development of cardiovascular and cerebrovascular diseases. However, the molecular mechanism underlying this phenomenon is poorly understood. The present study was designed to investigate the effects of REM sleep deprivation on blood pressure in rats and the underlying mechanisms of these effects. After Sprague-Dawley rats were subjected to REM sleep deprivation for 5 days, their blood pressures and endothelial function were measured. In addition, one group of rats was given continuous access to L-arginine supplementation (2% in distilled water) for the 5 days before and the 5 days of REM sleep deprivation to reverse sleep deprivation-induced pathological changes. The results showed that REM sleep deprivation decreased body weight, increased blood pressure, and impaired endothelial function of the aortas in middle-aged rats but not young rats. Moreover, nitric oxide (NO) and cyclic guanosine monophosphate (cGMP) concentrations as well as endothelial NO synthase (eNOS) phosphorylation in the aorta were decreased by REM sleep deprivation. Supplementation with L-arginine could protect against REM sleep deprivation-induced hypertension, endothelial dysfunction, and damage to the eNOS/NO/cGMP signaling pathway. The results of the present study suggested that REM sleep deprivation caused endothelial dysfunction and hypertension in middle-aged rats via the eNOS/NO/cGMP pathway and that these pathological changes could be inhibited via L-arginine supplementation.
Background	3	a.Sleep is essential for an individual's mental, emotional, and physiological well-being. Insufficient sleep is prevalent in the population and is associated with cardiometabolic health outcomes[1]. It has been shown that insomnia with objective short sleep duration is associated with a high risk for hypertension[2,3], and the pathophysiological mechanisms underlying this association may relate to inappropriate arousal due to the overactivation of stress system functions[3,4].The present study was designed to investigate the effects of REM sleep deprivation on blood pressure in rats and the underlying mechanisms of these effects. After Sprague-Dawley rats were subjected to REM sleep deprivation for 5 days, their blood pressures and endothelial function were measured. In addition, one group of rats was given continuous access to L-arginine supplementation (2% in distilled water) for the 5 days before and the 5 days of REM sleep deprivation to reverse sleep deprivation-induced pathological changes.<br>Six-week-old (young) and 24-week-old (middle-aged) male Sprague-Dawley rats were used， correspond to the youth and middle ages of the human.

		References<br>Altman NG, Izci-Balserak B, Schopfer E, Jackson N, Rattanaumpawan P, et al. Sleep duration versus sleep insufficiency as predictors of cardiometabolic health outcomes. Sleep Med. 2012;13: 1261-1270.<br>Vgontzas AN, Liao D, Bixler EO, Chrousos GP and Vela-Bueno A. Insomnia with objective short sleep duration is associated with a high risk for hypertension. Sleep. 2009;32: 491-497.<br>Li Y, Vgontzas AN, Fernandez-Mendoza J, Bixler EO, Sun Y, et al. Insomnia with physiological hyperarousal is associated with hypertension. Hypertension. 2015;65: 644-650.<br>Palagini L, Maria Bruno R, Gemignani A, Baglioni C, Ghiadoni L, et al. Sleep Loss and Hypertension: A Systematic Review. Curr Pharm Des. 2013;19: 2409-2419.
Objectives	4	Sleep loss can induce or aggravate the development of cardiovascular and cerebrovascular diseases. However, the molecular mechanism underlying this phenomenon is poorly understood. The present study is to understood the molecular mechanism.
METHODS		
Ethical statement	5	All experimental procedures were conducted in accordance with the National Institutes of Health Guide for the Care and Use of Laboratory Animals and were approved by the ethics committee of Shanghai University of Traditional Chinese Medicine.
Study design	6	a.The number of experimental group or control group was six.<br>b.Experimental animals were randomly divided into groups.<br>c.All of the animals were housed in individual cages on a 12 h light-dark cycle in a room with temperature and humidity control and were allowed access to standard rat chow and distilled water ad libitum.
Experimental procedures	7	Six-week-old (young) and 24-week-old (middle-aged) male Sprague-Dawley rats were purchased from Shanghai Slack Laboratory Animal Co., Ltd. (Shanghai, China). All of the animals were housed in individual cages on a 12 h light-dark cycle in a room with temperature and humidity control and were allowed access to standard rat chow and distilled water ad libitum. After 1 week of accommodation to environmental conditions.Animals were used for experiments.animals were continuously kept on a small raised platform (with a diameter of 6.0 cm for young rats and 6.5 cm for middle-aged rats) surrounded by water up to 1 cm beneath the platform surface for 5 days. When they reached the paradoxical phase of sleep, muscle atonia caused them to fall into the water and awaken. Control rats were maintained on a larger platform (with a diameter of 15 cm) in a similar environment. Rats supplemented with L-arginine were continuously provided with access to L-arginine (2% in distilled water) for 5 days before REM sleep deprivation. Subsequently, these animals were continuously provided with access to L-arginine or vehicle (distilled water) for the 5 days of REM sleep deprivation. Food and

		water were available ad libitum through a grid placed atop the water tank.
Experimental animals	8	Six-week-old (young) and 24-week-old (middle-aged) male Sprague-Dawley rats were purchased from Shanghai Slack Laboratory Animal Co., Ltd. (Shanghai, China).
Housing and husbandry	9	All of the animals were housed in individual cages on a 12 h light-dark cycle in a room with temperature and humidity control and were allowed access to standard rat chow and distilled water ad libitum.
Sample size	10	The number of experimental group or control group was six.
Allocating animals to experimental groups	11	Six-week-old (young) and 24-week-old (middle-aged) male Sprague-Dawley rats were purchased from Shanghai Slack Laboratory Animal Co., Ltd. (Shanghai, China). Six-week-old (young) rats were randomly divided into two groups:Control group and REMSD group(n=6).24-week-old (middle-aged) were randomly divided into three groups:Control group 、 REMSD group and REMSD+ L-arginine group(n=6).
Experimental outcomes	12	1)REM sleep deprivation caused significant reductions in body weight in young and middle-aged rats . This sleep deprivation also increased the blood pressure of middle-aged rats, with increases of 16% and 18% in systolic and diastolic blood pressure, respectively . However, the blood pressure of young rats was not changed after 5 days of sleep deprivation.<br>2)REM sleep deprivation did not affect ACh-mediated vasodilatation in the aortas of young rats. However, the endothelial function of middle-aged rats was significantly damaged by REM sleep deprivation.<br>3)REM sleep deprivation decreased NO production and cGMP levels in the aortas of middle-aged rats.<br>4)Western blotting revealed decreased expression of the phosphorylated eNOS protein in REM sleep-deprived rats. However, the overall level of eNOS protein was not significantly affected by REM sleep deprivation.<br>5)The results showed that L-arginine decreased blood pressure, improved NO-mediated vasorelaxation, raised NO and cGMP levels in the aorta , and increased eNOS phosphorylation .
Statistical methods	13	All data were expressed as means±SEM, and n refers to the number of rats. SPSS 18.0(SPSS Inc, Chicago, IL, USA) was used for comparisons of multiple groups via one-way analysis of variance (ANOVA) and LSD tests. Differences were considered statistically significant if $P<0.05$.
RESULTS		
Baseline data	14	Six-week-old (young) and 24-week-old (middle-aged) male Sprague-Dawley rats.The weigh of middle-aged 558±26g.
Numbers analysed	15	The number of experimental group or control group was six.
Outcomes and estimation	16	All data were expressed as means±SEM.

Adverse events	17	No
		DISCUSSION
Interpretation / scientific implications	18	The present study showed that REM sleep deprivation damaged the eNOS/NO/cGMP signaling pathway and caused endothelial dysfunction and hypertension. L-arginine supplementation could protect against these changes induced by REM sleep deprivation; this finding might give rise to a new interventional strategy for sleep loss-induced hypertension or other cardiovascular diseases.The present results were consistent with clinical studies indicating that a short sleep duration was associated with an increased risk of hypertension in middle-aged and elderly subjects, suggesting that elderly people are particularly vulnerable to sleep loss or insomnia.The present study provides a new strategy for inhibiting the signaling pathways that contribute to insomnia-induced or insomnia-enhanced cardiovascular diseases.
Generalisability / translation	19	A new strategy for inhibiting the signaling pathways that contribute to insomnia-induced or insomnia-enhanced cardiovascular diseases.
Funding	20	The present study was supported by the Young Research Foundation of Shanghai Health Bureau (20144Y0152), the Shanghai Education Budget Foundation (2015YSN25), and the Scientific Research Foundation for Returned Overseas Chinese Scholars, China State Education Ministry (N138590).
